# Talon Cusp Type I: Restorative Management

**DOI:** 10.1155/2015/425979

**Published:** 2015-05-06

**Authors:** Rafael Alberto dos Santos Maia, Wanessa Christine de Souza-Zaroni, Raul Sampaio Mei, Fernando Lamers

**Affiliations:** ^1^Oral and Maxillofacial Surgery, HGU, University of Cuiabá, 78016-000 Cuiabá, MT, Brazil; ^2^School of Dentistry, Cruzeiro do Sul University (UNICSUL), 08060-070 São Paulo, SP, Brazil; ^3^School of Dentistry, University Center of Grande Dourados (UNIGRAN), 79824-900 Dourados, MS, Brazil

## Abstract

The teeth are formed during intrauterine life (i.e., gestation) during the odontogenesis stage. During this period, the teeth move until they enter the oral cavity. This course covers various stages of dental development, namely, initiation, proliferation, histodifferentiation, morphodifferentiation, and apposition. The talon cusp is an anomaly that occurs during morphodifferentiation, and this anomaly may have numerous adverse clinical effects on oral health. The objective of this study was to report a case of “Talon Cusp Type I” and to discuss diagnostic methods, treatment options for this anomaly, and the importance of knowledge of this morphological change among dental professionals so that it is not confused with other morphological changes; such knowledge is required to avoid unnecessary surgical procedures, to perform treatments that prevent caries and malocclusions as well as enhancing aesthetics, and to improve the oral health and quality of life of the patient.

## 1. Introduction

Aetiology for the formation of the talon cusp is unknown. However, this may be due to the combination of genetic and environmental factors and hyperactivity of the dental lamina early in odontogenesis [1].

This anomaly is characterized by an accessory cusp as a projection of the cingulate area near the cementoenamel junction that is present in anterior teeth and is attached to the lingual surface, which follows the long axis of the tooth crown. This leaflet can vary in shape and length. This disorder is more common in maxillary incisors than in the jaw and can occur in both dentitions; it is most prevalent in the upper lateral incisors, followed by the maxillary central incisors [2].

The first description of this “accessory cusp” dental anomaly was based on the cingulate incisor side of a central incisor. Nearly a century later, the nomenclature was changed to “talon cusp” (talon refers to claw) because the anomaly presented with morphological characteristics similar to an eagle talon. Since then, the odontogenic abnormality has received several other classifications, such as exaggerated cingulate, additional cusp, accessory cusp, and supernumerary cusp [3].

Oredugba [4] reported that this change presents as altered enamel and dentin composition with a variable amount of pulp tissues. It is difficult to establish pulp involvement due to the overlap of the talon, tooth crown, and main pulp chamber in the radiographic image. Although some authors have found pulp communication to the talon cusp, others have reported no evidence of pulp extension on the cusp. However, it has been suggested that cases of large jaw cusps, especially those that fully protrude out of the tooth crown, are more likely to contain pulp tissue [5].

Radiographically, when the talon is located in the maxilla, it is characterized by a “V”-shaped structure with greater radiopacity in the dental crown. Characteristically, the talon or semituber comes from the cervical third of the tooth crown. The higher radiopacity of this structure should overlap with the “V” structure on the crown of the tooth image. In cases that present with a characteristic inverted “V” in the jaw, there may be overlapping images of features described in the maxilla. This appearance may vary in shape and size based on the angle at which the ray is taken [6].

The anomaly varies widely in form and size. Therefore, Hattab et al. [5] classified it into three groups: Type I: talon; Type II: semitalon; and Type III: trace talon or prominent cingulate. In Type I (talon), there is an additional cusp with prominent and well-defined morphological characteristics of a talon that projects from the lingual face of a front tooth (deciduous or permanent); the talon extends above half of the clinical crown of the tooth from the cementoenamel junction. Type II (semitalon) cases occur when an additional tip of a millimeter or more extends over less than half of the dental crown from the cementoenamel junction; these can blend with the palate surface or stand away from the rest of the crown. Type III (trace talon or prominent cingulate) cases present with a broad girdle or prominent appearance, and the variations include the conical type, bifid, and tuber.

The talon cusp is of unknown etiology, but it has been suggested that a combination of genetic and environmental factors is involved in its development. The most common explanation suggests that it develops in the morphodifferentiation stage; disturbances in this period, such as changes in endocrine function, can affect tooth shape and size without impairing the function of ameloblasts and odontoblasts [7]. Thirumalaisamy et al. [8] reported that, during morphodifferentiation, epithelial cell folding among the internal cells of the enamel (ameloblast precursors) and focal hyperplasia transiting the dental papilla mesenchymal (odontoblast precursors) result in the peculiar characteristic of a talon.

In addition to genetic influence, the talon cusp sometimes occurs alone or associated with other dental anomalies, such as mesiodens, odontoma, included or impacted teeth, cleft lips, nose wing distortion, bilateral twinning, merger, supernumerary teeth, and cracked enamel. It has also been associated with certain systemic conditions, including Mohr syndrome, Sturge-Weber syndrome, Rubinstein-Taybi syndrome, Bloch-Sulzberger syndrome, and Ellis-van Creveld syndrome [8].

The talon cusp results in complications related to four basic categories: diagnosis, function, aesthetics, and pathology. If the talon cusp is not diagnosed correctly and is confused with other pathologies, such as odontoma or supernumerary tooth, this may result in unnecessary surgery. Functionally, depending on the size of the talon, it can result in occlusal interference, accidental dental injury with possible pulp exposure, soft tissue injuries (such as to the tongue during speech or chewing), speech problems and tooth mobility due to premature contacts, and pain in the temporomandibular joints. Depending on the size of the leaflet and its location in the dental arch, the talon can be observed when a patient smiles or speaks, creating aesthetic complications. Because the deep grooves that connect the jaw to the tooth can retain plaque and food debris because cleaning is difficult, caries, subsequent periapical pathologies, and perhaps periodontal disease can subsequently develop [9].

Hattab et al. [5] reported that only the sealing of cracks is recommended for leaflets that do not have major clinical complications. If there is evidence of dental caries, the tooth must be restored. In cases in which the talon cusp creates a premature contact and occlusal interference, the authors recommended gradual reduction at consecutive visits over the course of 6–8 weeks. This period of reduction is important so that reparative dentin is deposited to protect the pulp. It is also important that a desensitizing agent is applied after each reduction session to prevent pain from the exposure of dentinal tubules.

According to Thirumalaisamy et al. [8], in the event of pulp exposure during the gradual reduction of the talon cusp, endodontic treatment that is best suited for the tooth in question should be performed. For this, the degree of development and root vitality should be noted, and the traditional endodontic technique, apexification, or the technique of apexogenesis should be utilized.

The differential diagnosis of this morphological change is important to identify the ideal treatment of the talon cusp. If not, complications such as pulpal or soft tissue lesions, speech problems, poor aesthetics, dental mobility and, in more severe cases, pain in the temporomandibular joints can occur. Therefore, the dentist must be careful not to confuse this particular anomaly with other changes, such as compound odontoma or supernumerary tooth, that can lead to incorrect diagnosis and inappropriate treatment [8].

Thus, the aim of this study is to report a case of “Talon Cusp Type I” and to discuss diagnosis methods, treatment options, and the importance of knowledge of this morphological change among dental professionals so that it is not confused with other morphological changes; such knowledge is required to avoid unnecessary surgical procedures, to perform treatments to prevent caries, and to improve malocclusions, aesthetic issues, oral health, and patient quality of life.

## 2. Literature Review

The cusp claw was initially described by Mitchell (1892) as an accessory cusp structured like a cingulate on the incisor side of a central incisor. The cusp claw was later named a talon cusp (talon refers to claw) by Mellor and Ripa [3] to signify the resemblance to an eagle talon. It is morphologically well defined and extends from the cementoenamel junction, reaching and sometimes exceeding the incisal edge. According to Hattab et al. [5], these cusps can be classified as Type I, II, or III.

Morphologically, the structure appears as an accessory cusp that protrudes from the region of the cingulate or cementoenamel junction to the anterior teeth, with contact on the lingual surface of the crown in the longitudinal direction. This structure varies in size, shape, length, and degree of contact with the lingual surface. There is a higher incidence in the maxilla than in the mandible.

There is no consensus among researchers regarding etiology; however, it has been suggested that a combination of genetic and environmental factors plays a role [4, 10–13].

This change occurs during the period of odontogenesis morphodifferentiation [6, 14]. This change may result in an imbalance in the stomatognathic system and subsequently present complications such as aesthetic problems, occlusal interferences, accidental dental trauma with a high possibility of pulp exposure, caries, periodontal problems, irritation of soft tissue during speech or chewing, and exacerbated pain in the temporomandibular joints [10–13].

Hattab et al. [5] recommend that, in cases of Talon Cusp Type III, which does not present with major clinical complications, only sealing of developmental grooves should be performed. If there is evidence of dental cavities, the decayed tissue should be removed, and conventional restorative treatment should be provided. However, if the talon cusp presents with occlusal interferences, usually Type I, the authors recommended a gradual reduction procedure in 6- to 8-week intervals to stimulate the deposition of reparative dentin and for pulp protection; this procedure should be accompanied by the use of a dentinal desensitizer. In cases of pulp involvement, Thirumalaisamy et al. [8] stated that the endodontic treatment best suited for the tooth in question should be performed based on the degree of root development and pulp vitality.

## 3. Case Report

**Figure 1:**
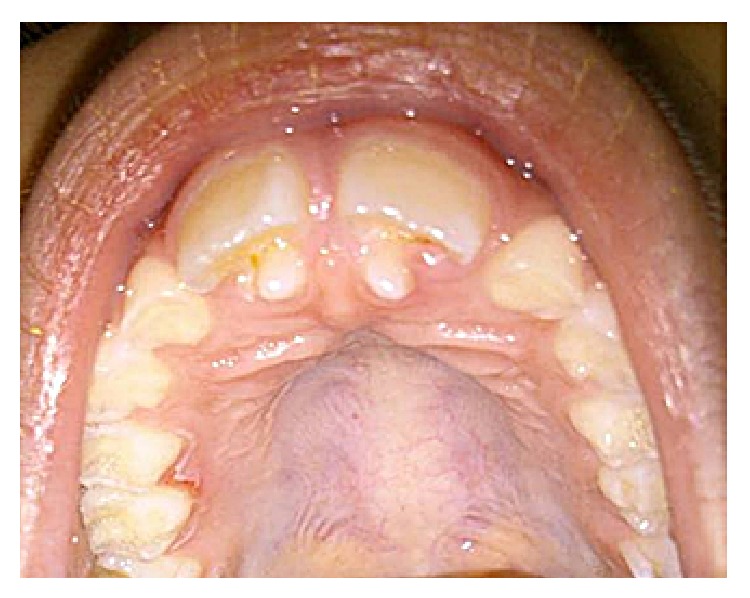
Anomalous projection elements in the region of 11 : 21.

Patient XX, 8 years old, attended the Children's Dental Clinic Multidisciplinary University of Grande Dourados Center (UNIGRAN) with his legal representative, who reported that it “looked like a tooth was rising behind the front teeth” (Figure 1).

**Figure 2:**
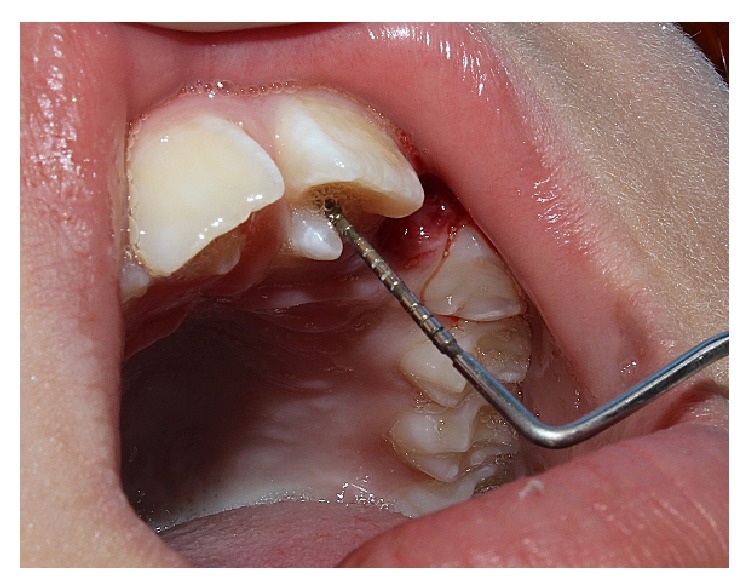
Cusp talon 6 mm in height with a probing depth of 1-2 mm.

After authorization by signatures on the Informed Consent Agreement from the legal guardian and the patient, a clinical examination was performed. An anomalous projection was observed in the cingulate region of elements 11 and 21 (upper central incisors); the projection was 6 mm high with a 1-2 mm groove area of probing depth (Figure 2).

**Figure 3:**
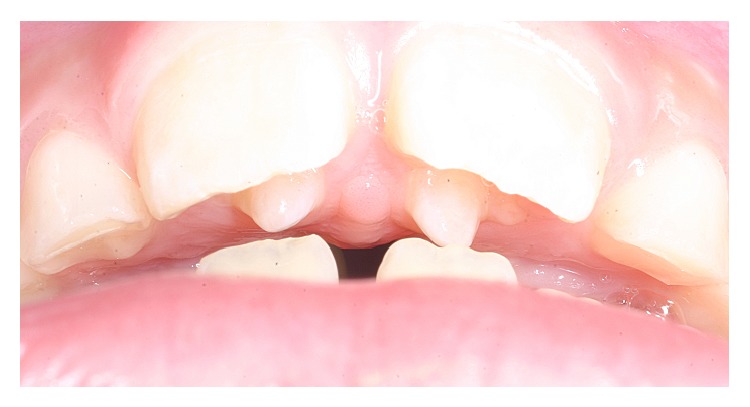
Presence of occlusal interference and premature contacts with the antagonist teeth.

The projection presented with Type I cusp features; it was an additional cusp of altered morphology with a well-defined prominence that featured a talon protruding from the lingual extension, with more than half of the clinical crown of the tooth extending to the cementoenamel junction. The groove had retained biofilm and food waste due to cleaning difficulty, but there was no trace of carious processes. Subsequently, it was observed that the size of the anomaly created an occlusal interference and premature contacts with the antagonist teeth (permanent lower central incisors), which had first-degree mobility (1 mm towards VL and MD; Figure 3).

**Figure 4:**
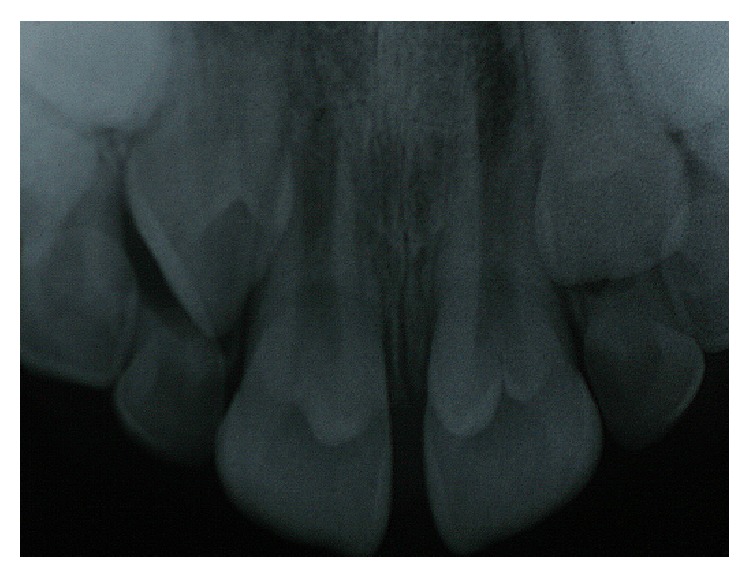
Radiographic appearance of the talon cusp.

Radiographically, there was a structure in the shape of a “V” with greater radiopacity in the tooth crown, which had characteristics similar to a cusp originating from the cervical third of the teeth. It was hypothesized that this higher radiopacity was the result of replacing the “V” structure on the image of the clinical crown of the tooth, which, combined with the clinical examination, was diagnosed as “leaflet of Type I talon.” It was not possible via radiographic examination to establish whether there was pulp involvement with the talon cusp (Figure 4).

According to Hattab et al. [5], the treatment protocol in cases of premature contact and occlusal interference involves gradual reduction over a 6–8-week period to stimulate reparative dentin deposition and to promote pulp protection while avoiding exposure of dentinal tubules that cause pain. However, we opted for the radical treatment reported by Ozcelik and Atila [14]; the two leaflets were reducted in a single session using a diamond cutter with high-speed intermittent movement and cooling while the patient was properly anesthetized. The option for radical treatment was chosen because of the presence of first-degree tooth mobility and chronic occlusal interference with the antagonist teeth, thereby aiming to reduce this mobility and restore the occlusal balance.

**Figure 5:**
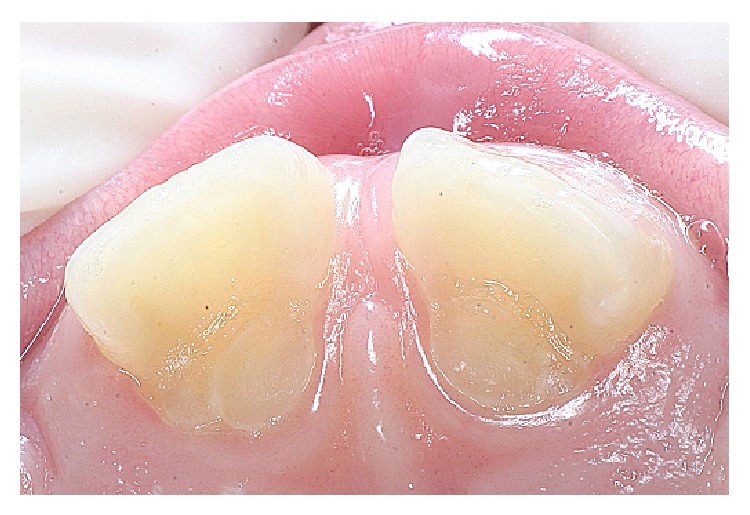
Final aspect of treatment.

After complete removal of the leaflets did not create pulp exposure, desensitizing materials (Colgate Duraphat) were applied to block the exposure of the dentinal tubules, thereby generating no sensitivity to the patient. The groove regions were properly sealed with sealant pits and fissures (FluroShield Dentsply) that release fluoride (Figure 5).

**Figure 6:**
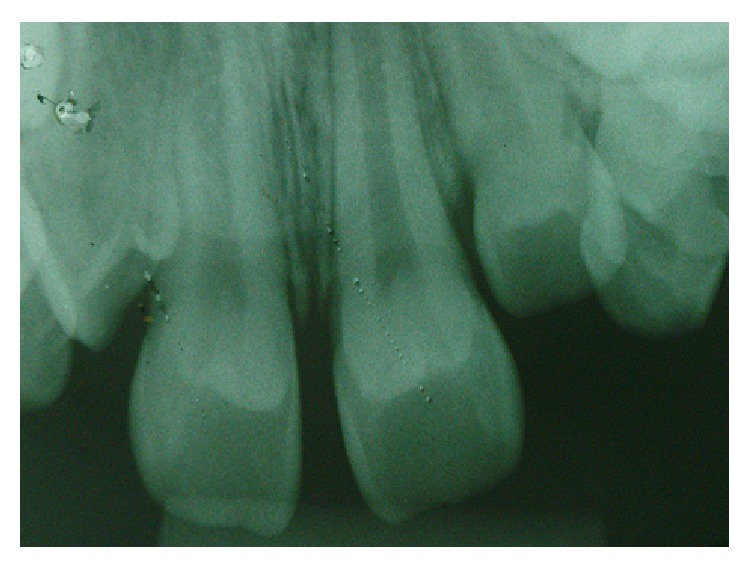
Final radiographic appearance.

Seven months after the first visit, the patient attended the Dental Clinic of UNIGRAN for a consultation, where we took X-rays that demonstrated the final treatment outcome: total reduction of the talon cusp without pulp involvement (Figure 6). There was no reported clinical dentinal sensitivity, the occlusal interferences were removed, and the food-retaining groove areas maintained a satisfactory seal.

## 4. Discussion

The development of human dentition occurs with one histophysiological pattern for each tooth germ. Each stage of dental development (bud stage or initiation; Hood or proliferation phase; Campanula phase or histodifferentiation; bell advanced stage, or morphodifferentiation; and root or affix phase) is susceptible to injury and dental anomalies. Specifically, in the morphodifferentiation step, the teeth can be affected by the talon cusp, which can be confused with other dental anomalies if a very thorough clinical and periapical examination is not performed [2]. This anomaly may be associated with supernumerary teeth or macrodontia invaginatus dens and is the result of hyperactivity of the dental lamina; there is a higher prevalence in the upper lateral incisors, followed by the maxillary central incisors, both in the permanent dentition. The etiology of this anomaly is not well defined and is considered to be multifactorial [15–17].

Davis and Brook [15] stated that knowledge of this anomaly is important for an accurate diagnosis of the cusp talon and to avoid unnecessary surgical procedures, such as tooth extractions. Furthermore, the diagnosis is important to prevent dental problems, such as caries development in the groove, aesthetic impairment, occlusal interference resulting from tooth displacement, speech problems, and soft tissue lesions (especially on the tongue). If caries lesions are present, the lesions are removed and restored; in cases of premature contact and occlusal interference, the talons should be reduced gradually [10, 12, 13].

According to Hattab et al. [5], grinding the talon cusp is recommended in cases with evidence of premature contact and occlusal interference; this procedure should be performed gradually in consecutive visits over a 6–8-week period to allow time for the deposition of reparative dentin and to protect the pulp. In contrast to this described protocol, Ozcelik and Atila [14] proposed to diminish the cusp talon in a single session; this protocol was termed a “radical treatment” approach and was adopted for the case presented in this paper due to the presence of tooth mobility and chronic occlusal interference with the antagonist teeth. As such, this procedure reduced the mobility and restored the occlusal balance.

It should be noted that, in the event of pulp involvement, both treatment protocols involving diminishing the talon cusp should include endodontic treatment best suited for the tooth in question, depending on the degree of root development and pulp vitality [8].

## 5. Conclusion

It is evident that, during the course of the dentition stage of development, anomalies can occur that create the talon cusp; this disorder occurs during the morphodifferentiation period. In the case of the Talon Cusp Type I, the morphologically altered cusp appears with a prominent and well-defined talon feature that protrudes from the palatal side of a front tooth (deciduous or permanent), and the talon extends above the halfway point on the clinical crown of the tooth from the cementoenamel junction. This type of anomaly may cause occlusal and aesthetic impairments and facilitate the development of caries in the area of the developmental grooves.

Therefore, it is necessary that dental professionals recognize all types of this anomaly so as not to confuse it with other morphological changes, thereby avoiding unnecessary surgical procedures. A correct diagnosis is necessary to prevent decay, malocclusion, and aesthetic issues, thus improving the oral health and quality of life of the patient. The patient will be monitored at regular sessions to assess the presence of dentinal sensitivity in the repaired region, to confirm that the occlusal interference was eliminated by reducing the talon cusp, and to ensure that the talon developmental groove region no longer retains biofilm and remains satisfactorily sealed with FluroShield Dentsply.
